# The Ankle Fleck Sign Indicating Fibular Avulsion of the Superior Peroneal Retinaculum

**DOI:** 10.5334/jbsr.2884

**Published:** 2022-10-10

**Authors:** Kris Mertens, Filip Vanhoenacker

**Affiliations:** 1 AZ Sint-Maarten and Catholic University of Leuven, BE; 2 AZ Sint-Maarten and University (Hospital) Antwerp/Ghent, BE

**Keywords:** peroneal retinaculum avulsion, computed tomography, magnetic resonance imaging, conventional radiography, ultrasound

## Abstract

**Teaching point:** A linear flake of bone at the posterolateral aspect of the distal fibula indicates avulsion of the superior peroneal retinaculum and warrants further investigation by dynamic ultrasound.

## Case History

**Figure 1:**
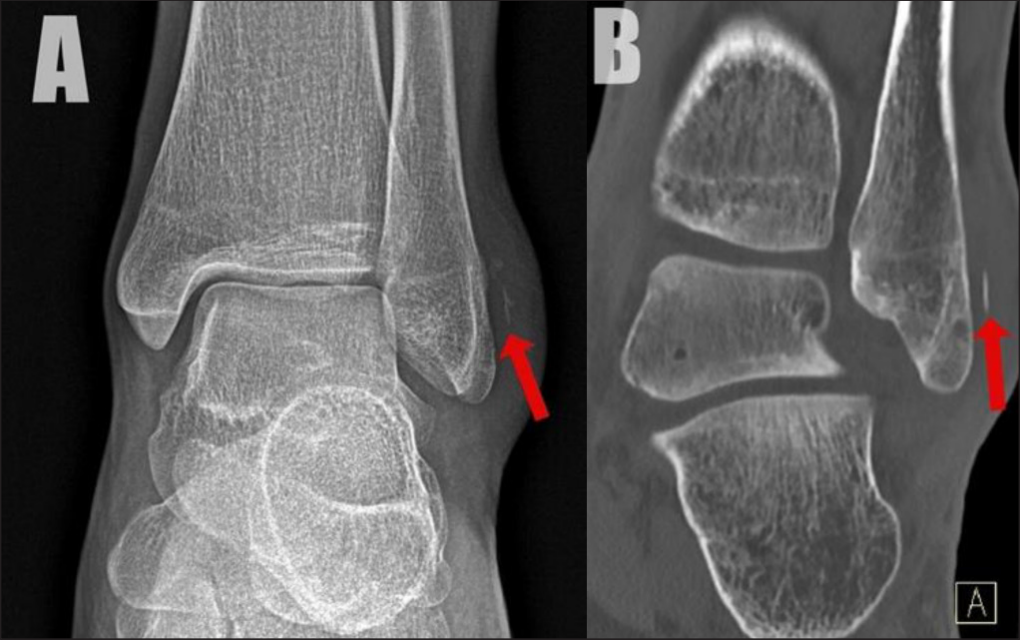
**A** and **B**.

**Figure 2:**
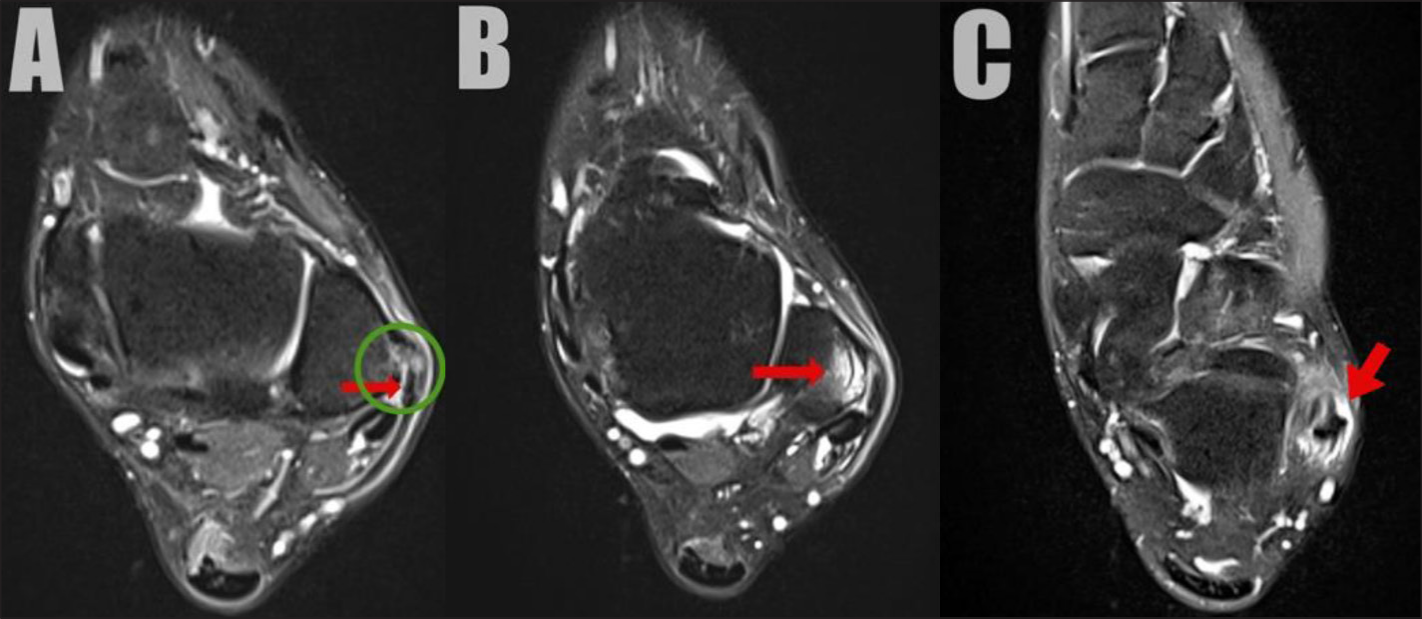
**A, B** and **C**.

**Figure 3:**
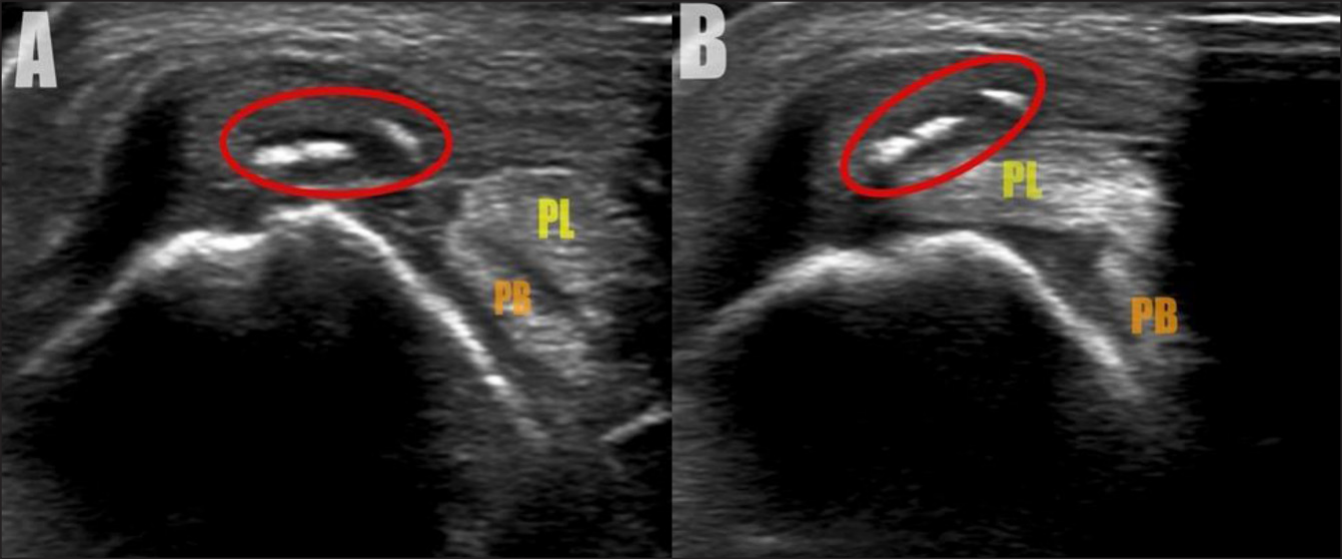
**A** and **B**.

A 22-year-old female presented with a persistent swelling at the lateral malleolus and limited range of motion after a sports injury two months earlier. Conventional radiography and computed tomography revealed a detached osseous fragment at the posterolateral cortex of the lateral malleolus associated with locoregional soft tissue swelling (Figure 1 A–B, AP radiograph and coronal bone window, arrow). Subsequent magnetic resonance imaging (MRI) confirmed the fragment located at the distal attachment of the superior peroneal retinaculum with retraction of the retinaculum (Figure 2A, red arrow and green circle, respectively). Fat suppressed (FS) T2-weighted images (WI) showed bone marrow edema in the distal posterolateral fibula (Figure 2B: red arrow). In addition, there was slightly increased fluid in the inframalleolar part of the sheath of the peroneus tendon (Figure 2C, red arrow). Ultrasound confirmed multiple small avulsion fragments along with perilesional edema at the peroneal retinaculum (Figure 3A–B, red circle; Video 3C). Palpation during dynamic ultrasound demonstrated a ventral subluxation of the peroneal longus tendon between the fragments and the fibula (Figure 3A–B, Video 3C; PL = peroneus longus, PB = peroneus brevis).

**Video 3C:** 

## Comment

The peroneal tendons both run in a common synovial sheath posteriorly to the lateral malleolus in the retro-malleolar groove where they are stabilized by the superior peroneal retinaculum. Injury of the peroneal retinaculum could lead to an increased friction of the tendons, tearing of the tendons and a higher risk of peroneal instability often resulting in spontaneous dislocation.

Patients mostly present with ecchymosis, swelling, pain and focal tenderness along the lateral malleolus.

In case of an avulsion fracture at the fibular insertion of the retinaculum, conventional radiographs show a flake of bone at the posterolateral distal fibula, designated as the ‘ankle fleck sign’.

On MRI, in the acute setting, rupture associated with edema of the retinaculum is seen on FS T2-WI. Scarring of the retinaculum with secondary tethering of the peroneal tendons and/or sural nerve can be seen in the chronic stage. Subluxation of the peroneal tendons is not always visible on static MRI but is best demonstrated during dynamic ultrasound imaging during dorsiflexion, eversion and/or sonopalpation [1]. The treatment of acute or chronic injury differs. Acute lesions are treated by plaster immobilization, whereas in chronic lesions reconstruction of the retinaculum and deepening of the fibular groove may be needed.
